# Computing limits on medicine risks based on collections of individual case reports

**DOI:** 10.1186/1742-4682-11-15

**Published:** 2014-03-24

**Authors:** Ola Caster, G Niklas Norén, I Ralph Edwards

**Affiliations:** 1 Uppsala Monitoring Centre, Box 1051, SE-751 40, Uppsala, Sweden; 2 Department of Computer and Systems Sciences, Stockholm University, Forum 100, SE-164 40, Kista, Sweden; 3 Department of Mathematics, Stockholm University, SE-106 91, Stockholm, Sweden

**Keywords:** Pharmacovigilance, Pharmacoepidemiology, Benefit-risk, Risk-benefit, Adverse drug reactions, Adverse drug reaction surveillance, Post-marketing, Post-marketing surveillance, Spontaneous reports

## Abstract

**Background:**

Quantifying a medicine’s risks for adverse effects is crucial in assessing its value as a therapeutic agent. Rare adverse effects are often not detected until after the medicine is marketed and used in large and heterogeneous patient populations, and risk quantification is even more difficult. While individual case reports of suspected harm from medicines are instrumental in the detection of previously unknown adverse effects, they are currently not used for risk quantification. The aim of this article is to demonstrate how and when limits on medicine risks can be computed from collections of individual case reports.

**Methods:**

We propose a model where drug exposures in the real world may be followed by adverse episodes, each containing one or several adverse effects. Any adverse episode can be reported at most once, and each report corresponds to a single adverse episode. Based on this model, we derive upper and lower limits for the per-exposure risk of an adverse effect for a given drug.

**Results:**

An upper limit for the per-exposure risk of the adverse effect Y for a given drug X is provided by the reporting ratio of X together with Y relative to all reports on X, under two assumptions: (i) the average number of adverse episodes following exposure to X is one or less; and (ii) adverse episodes that follow X and contain Y are more frequently reported than adverse episodes in general that follow X. Further, a lower risk limit is provided by dividing the number of reports on X together with Y by the total number of exposures to X, under the assumption that exposures to X that are followed by Y generate on average at most one report on X together with Y. Using real data, limits for the narcolepsy risk following Pandemrix vaccination and the risk of coeliac disease following antihypertensive treatment were computed and found to conform to reference risk values from epidemiological studies.

**Conclusions:**

Our framework enables quantification of medicine risks in situations where this is otherwise difficult or impossible. It has wide applicability, but should be particularly useful in structured benefit-risk assessments that include rare adverse effects.

## Background

Hardly any medicine is risk-free. Some undesirable effects are identified during pre-clinical and clinical testing, for regulatory benefit-risk assessments to decide whether or not the medicine should be granted market access. However, because pre-marketing trials are too small, too short, and too homogeneous with respect to included patients, many adverse effects remain undetected at the time of marketing [1]. Unless one is willing to rely on subjective expert guesswork, any quantitative evaluation of adverse effects from drugs requires data to inform risk estimation. This applies to analyses of single harmful effects and full-scale benefit-risk assessments alike. However, in reality rare adverse effects are very difficult to quantify [2]. Contrary to common belief, not only controlled clinical trials but also epidemiological studies in very large populations are likely to be underpowered for many important adverse effects [3]. Also, the standard epidemiological approach in situations of rare outcomes, the case-control study, can only estimate relative risk, which is of limited value in the decision-oriented context of benefit-risk assessment [4].

Pharmacovigilance is the over-arching scientific and regulatory discipline working to uncover possible new risks with medicines. In this process, the collection and analysis of individual case reports of suspected harm from medicines is a crucial component [5]. These reports are collected continuously in most countries worldwide, cover all types of drugs, and often contain detailed information to assist in the assessment of a possible causal link between drugs and adverse effects. It has also been argued that they can provide some quantitative risk information: because the number of reports on a particular drug-adverse event pair is lower than the actual number of affected patients due to under-reporting, dividing the report count by the number of exposed patients yields a lower limit on the true risk in these patients [6].

Following a recent update of regulatory post-marketing guidelines, companies are expected to complement the detection of a previously unknown and significant adverse effect with a complete benefit-risk assessment [7]. The academic community has contributed with a multifaceted development of quantitative approaches, which are expected to increase the transparency and consistency of benefit-risk assessment [8]. Regulators now express a clear interest in such methods, while companies remain more sceptical [9].

The aim of this work is to explore the boundaries for individual case reports as a source of information on the risk of adverse events under exposure to drugs. We present a conceptual model for the link between reported adverse events, real-world occurrences of adverse events, and exposed patients. We show how this model can be used to incorporate and formalise the above idea on a lower risk limit, and – most significantly – we derive from it an upper risk limit that is not dependent on external exposure data.

## A model for linking individual case reporting to the real world

To be able to explain how collections of individual case reports can provide quantitative risk information, we need to define a model that relates events reported as suspected adverse drug reactions to all such adverse events occurring in the real world. We start by a very brief description of individual case reports as such, followed by definitions of the core concepts necessary to understand the model, and then preceed to the actual model itself.

### Individual case reports

The origin of individual case reporting is so called spontaneous reporting of adverse drug reactions, with an explicit suspicion of drug attribution [10]. Today regulatory requirements are stricter and solicited reporting occurs in parallel to spontaneous reporting. Another recent phenomenon is that not only healthcare professionals but also patients themselves can report.

A majority of countries have national databases of individual case reports. One example is the Food and Drug Administration in the USA. There are also a few databases that cover several countries, e.g. the WHO global database of individual case safety reports, VigiBase [11]. In addition, all pharmaceutical companies have internal databases that cover their specific products.

All individual case reports must list one or several drugs as suspected, by themselves or in interaction, of having caused one or more adverse events; in addition, concomitant drugs may be listed. Drugs and events are typically coded in dedicated terminologies.

Much more information can be provided on the reports, including indication for use and start and stop dates for drugs, and dates for onset and abatament of events. Other important report fields include the reason for being classified as serious (if any); the dose and route of administration for drugs; and outcome for events.

The results of dechallenge, i.e. withdrawal of a drug, as well as rechallenge, i.e. re-exposure to the same drug, can be specified. Characteristics of the patient, such as age, gender, and medical history should also be provided. Finally, whereas all of the above is coded in structured format, one always has the opportunity to give a free-text description of the case.

### Exposure

Here, an *exposure* is to be understood as an episode of treatment with a given drug in an individual patient. Drugs, in turn, could refer to either substances or medical products, including or excluding vaccines and medical devices. It follows that disparate treatment episodes of the same patient with the same drug are considered as distinct and non-overlapping exposures. Also, the duration of an exposure can range from close to zero (e.g. a single bolus injection) to many years (e.g. life-long maintenance therapy for an incurable disease).

This definition is motivated by the nature of individual case reports, where one typically reports only those drugs that are currently being used or that were recently used by the patient, i.e. the currently relevant exposures. More flexible definitions of exposure are difficult, since patients are not followed continuously over time. For example, different treatment episodes within the same patient are not linked.

### Adverse episodes

An *adverse episode* is here defined as a set of clinical signs and symptoms that occur in an individual patient after exposure to one (or several) drugs. The signs and symptoms are clustered temporally and clinically so that they form an entity that corresponds to a single individual case report, if at all reported. It is therefore implied that an adverse episode can consist of one or several adverse events, which conforms with the appearance of individual case reports in reality.

This definition is very general and not restricted with respect to the time from start of exposure to onset of the adverse episode, nor with respect to the nature of the signs and symptoms contained in the episode. However, such restrictions can be reasonable or even necessary (see Sections ‘Low adverse episode density’ and ‘Using a restricted time to event onset’).

### Risk

The concept of *risk* as used here is the probability of experiencing a certain adverse event *Y* of interest after exposure to a certain drug *X* of interest, i.e. Pr(*Y*|*X*). This probability is given by the incidence of *Y* after exposure to *X*: in its most general form, the risk *r*_*xy *_is the fraction of all exposures to *X* that are followed by at least one occurrence of *Y*. If there is a requirement that *Y* occur within a certain time period *t* from start of exposure to *X*, this is indicated as $r_{\text{xy}}^{t}$. Unless otherwise specified, the risk applies to the entire population at issue.

### Model description

Table 1 defines the basic components of our model. Generally, the superscripts R, A, and E are used for reports, adverse episodes, and exposures, respectively. Two fundamental assumptions are that each adverse episode is reported at most once, and that each report describes an adverse episode that actually has occurred. These assumptions would be violated in the presence of report duplication, drug miscoding, or adverse event misdiagnosis. All are real but generally manageable threats in practice; for elaboration see the Section ‘General validity of the model’.

**Table 1:** Components of our linking model between individual case reporting and the real world

**Variable**	**Type of entities being counted**	**Context**
$N_{x}^{R}$	Reports on *X*	Database
$N_{\text{xy}}^{R}$	Reports on *X* together with *Y*	Database
$N_{x}^{A}$	Adverse episodes that follow exposure to *X*	Real world
$N_{\text{xy}}^{A}$	Adverse episodes that follow exposure to *X* and that include *Y*	Real world
$N_{x}^{E}$	Exposures to *X*	Real world
$N_{\text{xy}}^{E}$	Exposures to *X* followed by at least one adverse episode that includes *Y*	Real world

*X* and *Y* are the drug and adverse event of interest, respectively.

The quantity of main interest is the population risk *r*_*xy*_, defined above as the proportion among all exposures to *X*, $N_{x}^{E}$, that is made up by exposures to *X* that are followed by *Y*, $N_{\text{xy}}^{E}$:

(1) $$r_{\text{xy}}=\frac{N_{\text{xy}}^{E}}{N_{x}^{E}}\;.$$

Our aim is to relate this unknown risk to the known reporting ratio *ρ*_*xy *_between $N_{\text{xy}}^{R}$, the number of reports on *X* together with *Y*, and $N_{x}^{R}$, the total number of reports on *X*:

(2) $$\rho_{\text{xy}}=\frac{N_{\text{xy}}^{R}}{N_{x}^{R}}\;.$$

To be able to do so, we introduce the variables *f*_*x *_and *f*_*xy*_. They measure, respectively, the fraction of all adverse episodes following *X* that are actually reported, and the fraction of adverse episodes following *X* and containing *Y* that are actually reported:

(3) $$\begin{array}{lll} f_{x} & = & \frac{N_{x}^{R}}{N_{x}^{A}}\\ f_{\text{xy}} & = & \frac{N_{\text{xy}}^{R}}{N_{\text{xy}}^{A}} \end{array}\;.$$

**Figure 1:**
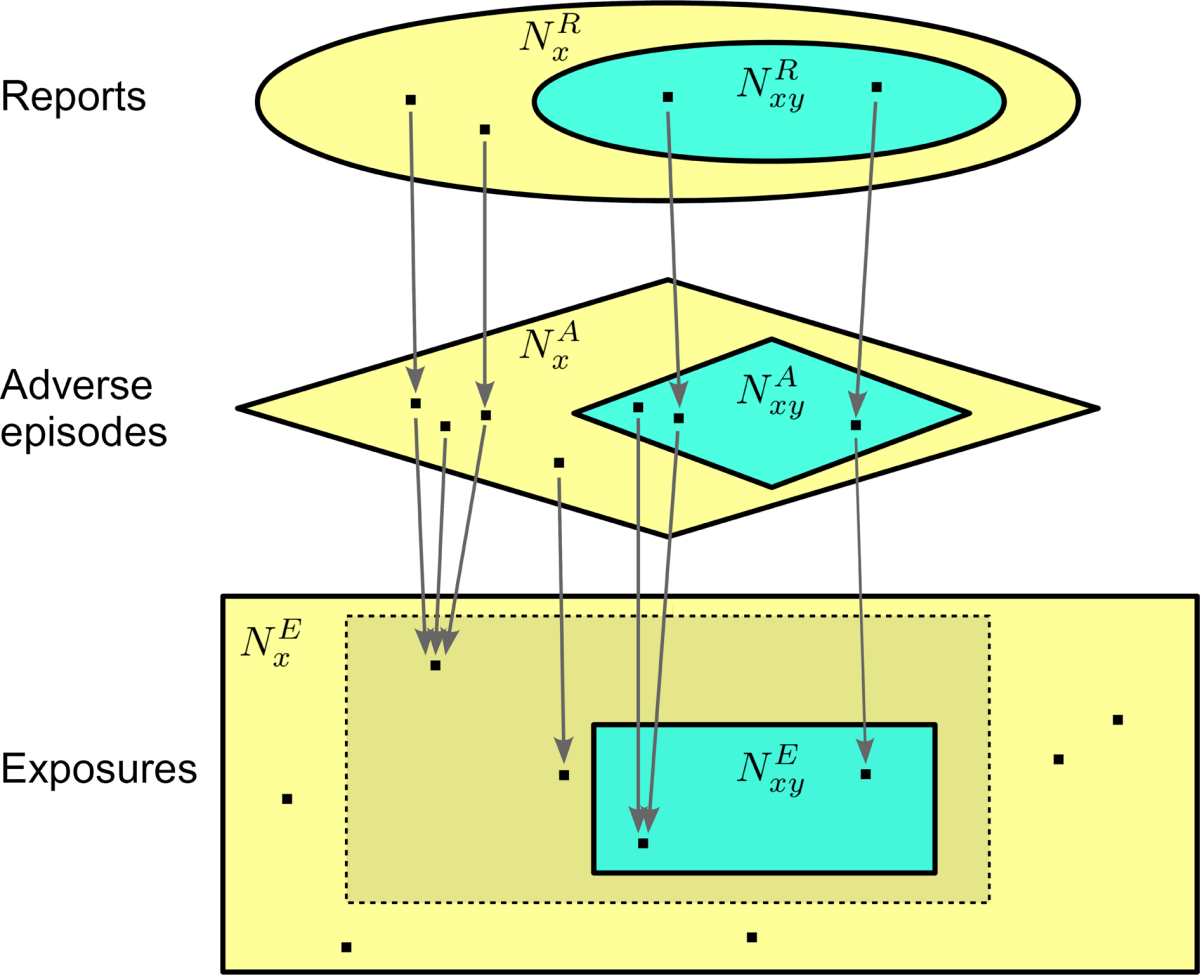
**Inter-component relations in our linking model between individual case reporting and the real world. **Note: All variables in the figure denote the numbers of elements of their respective sets, not the names of the sets themselves. Here, let the drug of interest *X* be ’analgesic’, and the adverse event of interest *Y* be gastrointestinal haemorrhage. The ellipses at the top represent the database of individual case reports: let $N_{x}^{R}$, the total number of reports on ’analgesic’, be 16,000, and assume that 400 of those reports concern gastrointestinal haemorrhage, i.e. $N_{\text{xy}}^{R}=400$. Thus, the reporting ratio for gastrointestinal haemorrhage with ’analgesic’ is *ρ*_*xy *_= 400/16,000 = 2.5*%*. Further, shapes with edges correspond to the real world: The rectangles represent the universe of exposures to ’analgesic’, and the diamonds represent the universe of adverse episodes that have followed those exposures. In this example, the total number of adverse episodes $N_{x}^{A}$ is 800,000, of which 5,000 contain gastrointestinal haemorrhage, i.e. $N_{\text{xy}}^{A}=5,000$. Each report maps to a single adverse episode, and each adverse episode is reported at most once: here the general reporting coverage for ’analgesic’ is $f_{x}=N_{x}^{R}/N_{x}^{A}=16,000/800,000=2\%$. The reporting coverage specifically for gastrointestinal haemorrhage with ’analgesic’ is $f_{\text{xy}}=N_{\text{xy}}^{R}/N_{\text{xy}}^{A}=400/5,000=8\%$. Those exposures that are followed by adverse episodes all reside within the dashed rectangle. Logically each of those exposures is mapped by at least one adverse episode, and each adverse episode maps to a unique exposure in the dashed rectangle. Of particluar interest are those exposures that are followed by at least one adverse episode containing gastrointestinal haemorrhage, i.e. the turquoise rectangle within the dashed rectangle. Here there are $N_{\text{xy}}^{E}=4,900$ such exposures, out of $N_{x}^{E}=1,000,000$ ’analgesic’ exposures in total. Hence, the true risk is $r_{\text{xy}}=N_{\text{xy}}^{E}/N_{x}^{E}=4,900/1,000,000=0.49\%$. All variables are described in Table 1.

We shall refer to *f*_*x *_and *f*_*xy *_as *reporting coverages*. (The commonly used quantity under-reporting is simply 1 - *f*.) All of the introduced concepts and their interrelations are outlined with a fictional example in Figure 1.

## Risk limits and their assumptions

Conditional on this model, we make two claims. First, the reporting ratio *ρ*_*xy *_is an upper limit for the risk *r*_*xy *_if (i) the total number of adverse episodes following exposure to *X* is less than or equal to the number of exposures itself; and (ii) the reporting coverage for adverse episodes that follow exposure to *X* and that include *Y* (*f*_*xy*_) is higher than or equal to the reporting coverage in general for adverse episodes following *X* (*f*_*x*_). These assumptions are clearly fulfilled for the example in Figure 1: (i) $N_{x}^{A}=800,000\leq 1,000,000=N_{x}^{E}$; and (ii) *f*_*xy *_= 8*% *≥ 2*% *= *f*_*x*_. As claimed, then, *ρ*_*xy *_= 2.5*% *≥ 0.49*% *= *r*_*xy*_. We shall refer to (i) as the assumption of *low adverse episode density*, and (ii) as the assumption of *relative over-reporting* of *Y* for *X*.

In general, the latter assumption should hold if *Y* is serious in nature, particularly if the link between *X* and *Y* is generally recognised or suspected. The former assumption should be more likely to hold the shorter the duration of treatment with *X*, and the healthier the population treated with *X*. However, there are possible countermeasures to apply in situations where this assumption is less likely to be valid. More elaboration is provided in the Section ‘Validity of the underlying assumptions’.

While a mathematical proof is provided below, *ρ*_*xy*_’s validity as an upper limit for *r*_*xy *_under these assumptions can be heuristically explained by considering the limiting case where each exposure is followed by precisely one adverse episode, so that $N_{x}^{A}=N_{x}^{E}$. Each adverse episode can then be considered an ‘observation’ of an exposure to *X*, and if those adverse episodes that contain *Y* are more likely to be reported than adverse episodes containing any adverse events, a greater proportion of ‘observations’ of *XY* end up in the database than ‘observations’ of *X* in general. If instead $N_{x}^{A}>N_{x}^{E}$, it could happen that *ρ*_*xy *_fell below *r*_*xy *_even if *f*_*xy *_≥ *f*_*x*_; for example, *Y* may occur only once per exposure in those exposures where it does occur, whereas there could be multiple adverse episodes per exposure in general, each with a possibility of being reported.

Secondly we claim that $N_{\text{xy}}^{R}/N_{x}^{E}$ is a lower limit for the risk *r*_*xy *_if the total number of reports on *X* with *Y* is fewer than or equal to the number of exposures to *X* that are followed by *Y*. This proviso, which will be referred to as *exposure-level under-reporting* of *XY*, is fulfilled for the example in Figure 1: $N_{\text{xy}}^{R}=400\leq 4,900=N_{\text{xy}}^{E}$. Here, the proposed lower limit is $N_{\text{xy}}^{R}/N_{x}^{E}=400/1,000,000=0.04\%$, which is below *r*_*xy *_= 0.49*%*. The lower limit requires knowledge about $N_{x}^{E}$ which is external to the database of individual case reports. Clearly, if $N_{x}^{E}$ is unknown, one can always report zero as the natural lower limit for *r*_*xy*_.

The assumption of exposure-level under-reporting should generally be valid. It basically serves to assure that the under-reporting of *Y* following *X* at the level of adverse episodes still holds at the level of exposures. This assumption would only ever be violated if *Y* recurred – and was reported – several times for individual exposures to *X*. However, it seems unlikely in practice that a recurring adverse event would be reported more than once in relation to the same exposure. Again, more details can be found in the Section ‘Validity of the underlying assumptions’.

### Proofs

For the upper limit, first note that the assumptions (i) and (ii) can be combined into $N_{x}^{A}/N_{x}^{E}\leq 1\leq f_{\text{xy}}/f_{x}$. This, in turn, is a special case of the more general condition $N_{x}^{A}/N_{x}^{E}\leq f_{\text{xy}}/f_{x}$, which is sufficient to guarantee that *ρ*_*xy *_provides an upper limit for *r*_*xy *_:

(4) $$\begin{array}{ll} \frac{N_{x}^{A}}{N_{x}^{E}}\leq \frac{f_{\text{xy}}}{f_{x}} & \Rightarrow \;\frac{N_{\text{xy}}^{E}}{N_{\text{xy}}^{A}}\frac{N_{x}^{A}}{N_{x}^{E}}\;\leq \;\frac{f_{\text{xy}}}{f_{x}}\\ & \Leftrightarrow \;\frac{N_{\text{xy}}^{E}}{N_{x}^{E}}\;\leq \;\frac{f_{\text{xy}}N_{\text{xy}}^{A}}{f_{x}N_{x}^{A}}\\ & \Leftrightarrow \;\frac{N_{\text{xy}}^{E}}{N_{x}^{E}}\;\leq \;\frac{N_{\text{xy}}^{R}}{N_{x}^{R}}\\ & \Leftrightarrow \;r_{\text{xy}}\;\leq \;\rho_{\text{xy}} \end{array}\;\;\;.$$

The implication on the top row holds because both $N_{x}^{A}/N_{x}^{E}$ and *f*_*xy*_/*f*_*x *_are positive, while by necessity $0<N_{\text{xy}}^{E}/N_{\text{xy}}^{A}\leq 1$ due to the nature of the mapping from adverse episodes to exposures explained in Figure 1. It is significant that this implication does not necessarily hold from right to left, which explains why *ρ*_*xy *_cannot easily be used as a lower limit for *r*_*xy*_. The equivalence on the third row follows directly from Equation 3, and that on the fourth row from Equations 1 and 2.

The validity of our claim regarding the lower limit for *r*_*xy *_can be trivially proven in the following way:

(5) $$\begin{array}{ll} N_{\text{xy}}^{R}\;\leq \;N_{\text{xy}}^{E} & \Leftrightarrow \;\frac{N_{\text{xy}}^{R}}{N_{x}^{E}}\;\leq \;\frac{N_{\text{xy}}^{E}}{N_{x}^{E}}\\ & \Leftrightarrow \;\frac{N_{\text{xy}}^{R}}{N_{x}^{E}}\;\leq \;r_{\text{xy}} \end{array}\;\;.$$

## Real-world examples

### Pandemrix and narcolepsy

The A/H1N1 influenza pandemic in 2009 was met by variable immunisation strategies. The two Nordic countries Sweden and Finland both launched mass-immunisation programmes with the specific vaccine Pandemrix. Suspicions arose in those countries regarding a causal relationship in children and adolescents between Pandemrix and the rare sleep disorder narcolepsy, a relationship with increasing support and acceptance [12-16].

The purpose of this example is to illustrate our framework. It was chosen on the basis that the exposure $N_{x}^{E}$ has been publicly reported in both Finland and Sweden, and that the immunisation coverage was substantive enough to enable epidemiological quantification of the risk *r*_*xy *_in these countries. Because of this quantification there is no apparent added value with our framework, which is most useful in situations where there is no other quantitative information available regarding the risk of interest. This was the case for Pandemrix and narcolepsy in August 2010.

#### ***At-risk patient population***

In this post-hoc setting, we will consider only those individual case reports that concern patients from the specific at-risk age groups that have been investigated epidemiologically in Finland in Sweden. This is to enable comparison between our derived risk limits and the reference values from the epidemiological studies, and need not reflect recommendable use of our approach in other settings. In Finland, the investigated cohort ranged from 4 to 19 years of age [12], while Swedish authorities noted an increased risk in patients aged 20 years or below [14].

#### ***Exposure***

In Finland, the number of Pandemrix vaccinees in the concerned age group has been reported to be precisely 688,566 [12]. The Swedish study was based on about 61% of the population, and in the age group 0-19 years the vaccination coverage was 69.5% [14]. Assuming that 69.5% of all Swedish 0- to 20-year-olds were vaccinated, and using demographic statistics from Statistics Sweden for the year 2009, $N_{x}^{E}$ for Sweden is estimated at 1.6 million.

In this case we have considered each vaccinee to have been exposed to a single treatment episode of Pandemrix regardless whether (s)he was given a single dose or two doses separated by a few weeks.

#### ***Time frame***

Both the Swedish and the Finnish Pandemrix immunisations were started in October 2009, with the former ending in March 2010 and the latter in August 2010 [12,14]. The initial suspicion of a potentially vaccine-induced life-long disability in previously healthy children and adolescents naturally triggered a lot of media attention. Because such attention can cause a dramatic increase of awareness and therefore reporting, we chose here to consider only reports submitted between 1^st^ October 2009 and 15^th^ August 2010, with narcolepsy coded. This end date was used also in the Finnish epidemiological study [12].

#### ***Risk limits and reference values***

**Figure 2:**
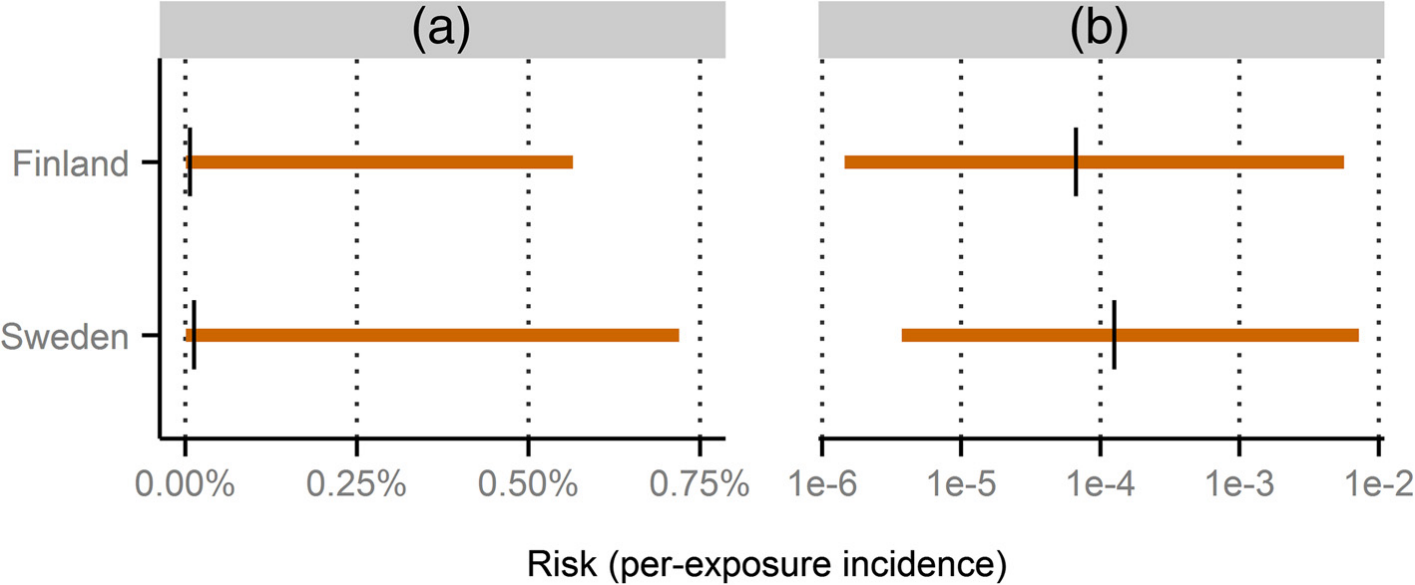
**Computed limits and reference values for the narcolepsy risk following Pandemrix vaccination. **The horizontal orange lines indicate the intervals computed as $\left[\;N_{\text{xy}}^{R}/N_{x}^{E};\;\rho_{\text{xy}}\right]$, and the black vertical lines indicate the reference values. Panel **(a) **shows the values untransformed, whereas panel **(b) **uses a logarithmic scale. All underlying data is presented in Table 2.

The number of individual case reports on Pandemrix in total – $N_{x}^{R}$ – and with narcolepsy in specific – $N_{\text{xy}}^{R}$ – were obtained directly from the Finnish and Swedish authorities^a^. All data used to compute the risk limits and the reference values are given in Table 2. The results are displayed in Figure 2.

**Table 2:** Data used to compute limits and reference values for the narcolepsy risk following Pandemrix vaccination

**Country**	**Age group**	$N_{\text{xy}}^{R}$ ^*^	$N_{x}^{R}$	$N_{x}^{E}$	**Reference risk**
Finland	4-19 years	1	177	688,566	46 cases in 688,566 vaccinees
Sweden	0-20 years	6^*‡*^	834	1.6 million^*†*^	126 cases in 1.0 million^*† *^vaccinees

*Reports with MedDRA preferred term ’Narcolepsy’.

^*‡ *^This excludes two cases reported before 15^th^ August 2010 but initially misdiagnosed.

^*‡ *^This is an estimate based on 69.5% vaccination coverage [14].

The reference risk values for Finland and Sweden were computed from the studies by Nohynek et al. [12] and Persson et al. [14], respectively.

In this example the upper and lower limits form intervals that contain the reference values obtained from the epidemiological studies, as intended. The intervals are wide for both countries, with the lower endpoints residing much more closely to the reference values than the upper endpoints, when viewed on an absolute scale. On a logarithmic scale, however, the reference values are located approximately on the intervals’ midpoints. This implies that the relative differences between the upper limits and the reference values are of the same magnitude as the relative differences between the reference values and the lower limits.

It should be noted that the higher Swedish reference value does not in itself imply a higher Pandemrix-attributable narcolepsy risk in Sweden compared to Finland. Because risk is defined as per-exposure incidence, one possible explanation to the higher value for Sweden is the substantially longer follow-up period used in the Swedish study compared to the Finnish.

### Antihypertensive drugs and coeliac disease

In 2011, the US Food and Drug Administration identified unexpectedly many reports for the angiotensin receptor blocker olmesartan together with the adverse event coeliac disease [17]. This discovery was followed by an investigation of the incidence of coeliac disease following use of olmesartan and several other antihypertensive drugs, as a so called modular program within the Mini-Sentinel project [18]. In Mini-Sentinel, data from several different collaborators are pooled together, which enables coverage of a fair proportion of the US population. In mid 2012 there were about 50 million patients enrolled with data on both drug usage and medical events [19]. Although this is not complete or near-complete population coverage as in the Pandemrix studies from the Nordic countries, it should be sufficient to provide reliable reference risk estimates for common drugs.

This particular example was chosen as the most recently completed modular program with an objectively identifiable adverse event: ‘Coeliac disease’ is a verbatim term both in the International Classification of Diseases used in Mini-Sentinel, and in the Medical Dictionary for Regulatory Activities (MedDRA) used for coding of individual case reports. As with Pandemrix and narcolepsy, the purpose of this example is to illustrate our framework. In particular, we do not intend to make any claims regarding causality. A potential benefit with this example is the minimal amount of public attention, especially for drugs other than olmesartan itself, which leaves little concern for potential reporting biases.

#### ***Included drugs***

To obtain accurate reference risk estimates, only drugs with at least 100,000 incident users in the Mini-Sentinel study were included: amlodipine, atenolol, hydrochlorothiazide, losartan, olmesartan, and valsartan [18].

#### ***Reference risk values***

The Mini-Sentinel report provides information on the number of incident users and incident events between 1^st^ January 2007 and 31^st^ December 2011 [18]. To match the definition of risk used here, it must be assumed that each user is exposed to a single treatment episode, and that each event belongs to a unique patient and therefore a unique exposure. These are reasonable assumptions given that antihypertensive drugs are used on a continuous basis, and that the event is rare.

#### ***Exposure***

For each included drug, the total number of exposures in USA between 1^st^ January 2007 and 31^st^ December 2011, $N_{x}^{E}$, was estimated based on the reported number of patients in the Mini-Sentinel cohort that were eligible for an incident treatment episode. The actual number of users in the cohort was scaled up to the entire population via the relation between the number of eligible patients and the total US population as of 30^th^ June 2009, according to the US Census Bureau.

#### ***Risk limits***

**Figure 3:**
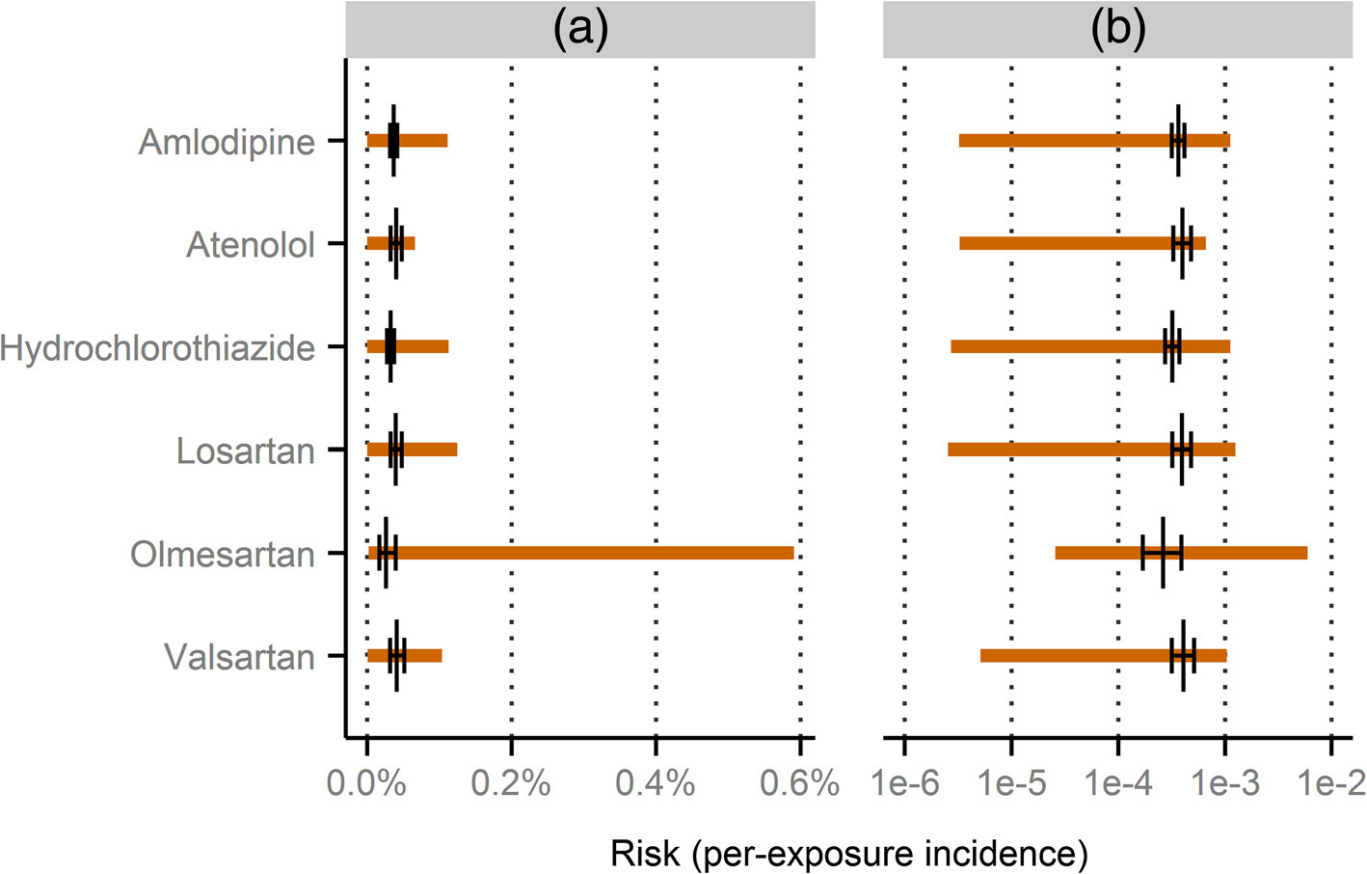
**Computed limits and reference values for the risk of coeliac disease following use of antihypertensive treatment. **The horizontal orange lines indicate the intervals computed as $\left[\;N_{\text{xy}}^{R}/N_{x}^{E};\;\rho_{\text{xy}}\right]$, and the black vertical lines indicate the reference point estimates with their corresponding 99% confidence intervals. Panel **(a)** shows the values untransformed, whereas panel **(b) **uses a logarithmic scale. All risks refer to the US population between 1^st^ January 2007 and 31^st^ December 2011. All underlying data is presented in Table 3.

The report counts required to compute the upper and lower risk limits for the six included drugs, $N_{x}^{R}$ and $N_{\text{xy}}^{R}$, were taken from the subset of VigiBase comprised by US reports with onset dates between 1^st^ January 2007 and 31^st^ December 2011. All data used to compute the risk limits and the reference values are given in Table 3, and the results are shown in Figure 3. The US subset of VigiBase is not identical to the national US database, and slight deviations in the results would be expected had the analysis been performed directly in the national database.

**Table 3:** Data used to compute limits and reference values for the risk of coeliac disease following antihypertensive treatment

**Drug**	$N_{\text{xy}}^{R}$ ^*^	$N_{x}^{R}$	$N_{x}^{E}$ ^***‡***^	**Reference risk**
Amlodipine	26	23,272	8.1 million	361 events in 991,184 users
Atenolol	12	18,166	3.7 million	181 events in 452,985 users
Hydrochlorothiazide	20	17,786	7.4 million	294 events in 913,563 users
Losartan	9	7,232	3.5 million	174 events in 440,583 users
Olmesartan	31	5,243	1.2 million	40 events in 151,461 users
Valsartan	12	11,603	2.3 million	118 events in 290,305 users

*Reports with MedDRA preferred term ’Coeliac disease’.

^*‡ *^This estimate is the actual number of users in the Mini-Sentinel cohort scaled up according to the number of eligible patients and the total number of US citizens.

The reference values were obtained from a Mini-Sentinel report [18], and the limits were computed based on US reports in VigiBase. All risks refer to the US population between 1^st^ January 2007 and 31^st^ December 2011.

The reference risk estimates for all six drugs, including their 99% confidence intervals, are contained between their corresponding upper and lower risk limits. Compared to the previous example, the reference values generally reside further towards the upper limits here. This is sensible considering that the Pandemrix example features a much shorter duration of treatment and a generally healthier population. (Cf. Section ‘Risk limits and their assumptions’ above). Olmesartan deviates from the other drugs by having considerably higher risk limits. This is unsurprising since the original concern was raised for this drug in particular, based on US individual case reports.

## Practical issues and possible developments

In this section we present and discuss some significant challenges and possible developments related to the practical application of our proposed framework.

### Validity of the underlying assumptions

A fundamental question in practice is whether the assumptions that predicate the calculation of the upper and lower limits are in fact valid. The following discussion summarises our collected view.

#### ***General validity of the model***

Our model presupposes that each adverse episode is reported at most once, and that each report describes an actual adverse episode from the real world. The former assumption would be violated if reports were duplicated, and the latter if adverse episodes were misrepresented when transferred to individual case reports. Because both phenomena do occur in practice it is clear that these assumptions are unrealistic; the real question is whether the nature and extent of their violations are manageable.

Theoretically, report duplication could invalidate our derived risk limits in two ways. It could artifically elevate $N_{x}^{R}$ to such an extent that the upper limit drops below *r*_*xy *_despite Equation 4 being fulfilled. This is unlikely since it would have to affect differentially those reports on *X* that do not include *Y*, and because the nominal level of duplication is low, typically below 5% [20]. Duplication could also artifically elevate $N_{\text{xy}}^{R}$ to a degree where the lower limit exceeds *r*_*xy *_despite Equation 5 being fulfilled. This too is unlikely given that the rate of duplication is likely to be far outweighed by the typical level of under-reporting, which is above 50% [21]. In addition, there are effective methods to identify suspected duplicates [20], which further minimises the practical significance of this issue.

When an adverse episode is misrepresented on a report, this report effectively has no corresponding adverse episode in the real world. Misrepresentations relevant for this framework are primarily miscoding of drugs, e.g. to mistake azathioprine for azacitidine, or misdiagnosis of adverse events, e.g. to report Stevens-Johnson syndrome for a common rash. Such errors create artificial reports and therefore pose the same kinds of threats as report duplication, but they simultaneously prevent actual adverse episodes from being properly reported, which has other consequences (see Section ‘Relative over-reporting of *Y* for *X*’ below). As with duplication, it is difficult to see how miscoding or misdiagnosis could happen on a significant enough scale to practically threaten the upper limit. However, for the lower limit these issues could be practically relevant, as indicated by the examples above: a single false report on a rare event like Stevens-Johnson syndrome when in reality there are no affected patients on the drug in question would invalidate the lower limit. Therefore, if the analysis is crucial and hinges on a small number of reports, it may be adviseable to validate the content of the reports with the original rapporteurs, if possible. This is particularly important if the event is difficult to diagnose, or if the drug is commonly confused with other drugs.

#### ***Exposure-level under-reporting of XY***

In the Section ‘Risk limits and their assumptions’ we introduced the assumption of exposure-level under-reporting of *XY* to guarantee that $N_{\text{xy}}^{R}/N_{x}^{E}$ provides a lower limit for *r*_*xy*_. This formally means that $N_{\text{xy}}^{R}/N_{\text{xy}}^{E}\leq 1$, so that the exposures to *X* that are followed by *Y* generate, on average, at most one report on *X* with *Y*.

If *Y* is rare or irreversible, then $N_{\text{xy}}^{E}$ is very close to $N_{\text{xy}}^{A}$, and the assumption essentially becomes $N_{\text{xy}}^{R}/N_{\text{xy}}^{A}=f_{\text{xy}}\leq 1$, which is true by definition in our model. However, even if *Y* is common and can recur several times after a single exposure to *X*, it should be very rarely reported more than once for an individual exposure. Therefore, given that under-reporting generally at the adverse episode level is at least 50%, it is difficult to see how the assumption of exposure-level under-reporting could be violated in practice.

On the contrary, if there is reason to assume that $N_{\text{xy}}^{E}/N_{\text{xy}}^{A}\approx 1$ and one can determine a value *fxy *′ such that *f*_*xy *_<*fxy *′ surely holds, then one can compute an improved lower limit for *r*_*xy *_as $\frac{N_{\text{xy}}^{R}/f_{\text{xy}}^{\prime }}{N_{x}^{E}}$.

#### ***Relative over-reporting of Y for X***

Two conditions were introduced that together imply the validity of *ρ*_*xy *_as an upper limit for *r*_*xy*_. One of these is relative over-reporting of *Y* for *X*, which states that *f*_*xy*_, the reporting coverage for adverse episodes following *X* and containing *Y*, should exceed *f*_*x*_, the reporting coverage for all adverse episodes following *X*.

The factors that influence reporting coverage are well studied [21,22]. Many of those factors relate to general attitudes among health care professionals and therefore have no impact on the relative values of *f*_*xy *_and *f*_*x*_. Similarly, factors that are related to the properties of *X* in general should not be of any concern, since they should affect *f*_*xy*_and *f*_*x*_ similarly. Rather our focus lies on factors that relate to the adverse event *Y*, possibly in relation to *X*.

If *Y* is such that its outcome is always or often serious, it is more likely to be reported [21]. In fact, it is a common misconception that *only* serious or severe reactions should be reported [22]. Therefore the assumption of relative over-reporting should hold generally for adverse effects that are serious in nature. This is fortunate, since serious and unusual effects are those of most importance after a medicine is marketed.

Common reasons for not reporting include diffidence and insecurity, i.e. fears of appearing foolish for proposing a controversial claim or not being able to provide sufficient evidence [22]. Therefore relative over-reporting should be more likely if the link between *X* and *Y* is commonly recognised or at least suspected.

Anomalous massive reporting on *X* with other events than *Y*, so called masking, could pose a threat to the assumption of relative over-reporting of *Y*, by increasing $N_{x}^{R}$ and therefore *f*_*x*_. Possible reasons include media attention or clustered reporting related to e.g. law suits. It is therefore adviseable to produce a general overview of the reporting on *X* to enable identification of apparent oddities.

Finally, if *Y* is difficult to diagnose or inconsistently coded, *f*_*xy *_could be differentially lowered in relation to *f*_*x*_ even if adverse episodes with *Y* are in fact more commonly reported than adverse episodes in general, after exposure to *X*. For example, neuroleptic malignant syndrome can be diagnosed and reported as such, but it is possible to instead report a subset of its constituent symptoms, either due to failure to recognise the syndrome, or failure to use the syndromic term. If one then computes *ρ*_*xy *_based only on the precise adverse event term ‘neuroleptic malignant syndrome’, lots of reports that actually describe adverse episodes with this syndrome will be excluded from the numerator. This effect can be mitigated by carefully defining *Y* within the adverse event terminology used in the database at hand.

#### ***Low adverse episode density***

The other assumption behind the upper limit states that $N_{x}^{A}/N_{x}^{E}\leq 1$, which means that the average number of adverse episodes per exposure – the so called adverse episode density – should be one or less. In practice, only those adverse events actually reported with *X* in the particular database at hand need to be considered when assessing the validity of this assumption, since none of the quantities involved in the calculation of the upper and lower limits are affected by exclusion of adverse events never reported with *X*.

Mainly two factors should influence the low adverse episode density assumption. The first is duration of treatment: the longer a drug is used, the more likely that some adversity occurs, whether related to the drug or not. The second is the nature of the underlying disease: the more serious the disease, the more likely that some disease-related symptom occurs in suspect temporality to drug intake. Also, more serious diseases such as cancer are often treated with drugs that have burdensome adverse effect profiles, which strengthens this effect. This implies that short-term treatments of healthy individuals, e.g. immunisations, are almost certain to withstand this assumption, whereas long-term treatments of seriously ill patients, e.g. lengthy chemotherapy regimens, are certain not to. More experience with this methodology is required to better understand how the low adverse episode density assumption works in less clearcut situations.

If the low adverse episode density assumption is unlikely to hold, it can be made more probable by exclusion of adverse events other than *Y*. Figure 1 provides a heuristic motivation: such an exclusion does not alter *r*_*xy*_, it merely shrinks the yellow diamond ($N_{x}^{A}$), which in turn causes the dashed yellow rectangle (the subset of $N_{x}^{E}$ followed by any adverse episode) and the yellow ellipse ($N_{x}^{R}$) to shrink. The more extreme the exclusion, the smaller $N_{x}^{R}$ becomes, and the larger *ρ*_*xy *_becomes. However, this method may affect other assumptions too. In particular, exclusion of common and non-serious events is likely to negatively influence the relative over-reporting of *Y*. Again, therefore, more practical experience is needed.

Another alternative is to consider only a specific time period *t* following start of exposure to *X*. As *t* is decreased, so is the probability of experiencing an adverse episode, and thereofore the density assumption is more and more likely to hold. This effect comes at the cost of calculating an upper limit for $r_{\text{xy}}^{t}$ rather than *r*_*xy*_, which may not be informative or interesting enough for the specific issue at hand.

As demonstrated in Section ‘Proofs’, the combination of relative over-reporting and low adverse episode density is an unnecessarily strong requirement: what matters is really that *f*_*xy*_/*f*_*x *_exceeds $N_{x}^{A}/N_{x}^{E}$, which may even be of practical relevance. However, the decomposition into two separate assumptions is very helpful to understand when and how *ρ*_*xy *_serves as an upper limit for *r*_*xy*_.

### Different types of risk

The total risk *r*_*xy *_considered thus far averages over all possible causes that may lead to adverse event *Y* after exposure to drug *X*. Alternative risks are possible, since if a new entity *Y*^′^ is defined as all occurrences of *Y* involving a specific cause, our model is still valid. The challenge in practice is to identify those individual case reports that describe occurrences of *Y*^′^, and it is generally necessary to retreat to conservative rules that treat $N_{\text{xy}}^{R}$ permissively for the upper limit and restrictively for the lower limit. Two examples are provided to illustrate this.

#### ***Total risk excluding the background related to other drugs***

One may be interested in those occurrences of *Y* that follow *X* and that are attributable either to *X* itself or to the general background, including e.g. the underlying disease but excluding other drugs. We label those events $\hat{Y}$ and their corresponding risk $\hat{r_{\text{xy}}}$.

While accurate identification of all reports on *X* with $\hat{Y}$ is difficult, the following is a tentative proposal: For the upper limit of $\hat{r_{\text{xy}}}$, exclude from $N_{\text{xy}}^{R}$ those reports where *X* is listed as concomitant and another drug is implicated as a cause; and for the lower limit, include only reports where *X* is sole suspected. The latter rule is clearly conservative, but so is the former: it selectively excludes reports where *Y* was caused by other drugs, and hence retains almost all reports where *Y* is attributable to *X* or the general background; at the same time, it is likely to erroneously retain quite a few reports where *Y* was caused by other drugs. Information that could implicate other drugs include positive dechallenge or rechallenge reactions, or an explicitly reported suspicion of a causal link.

#### ***Attributable risk***

In other settings, one’s main interest may be solely those occurrences of *Y* that have *X* on their causal pathway. Those events are denoted $\tilde{Y}$ with their corresponding risk $\tilde{r_{\text{xy}}}$, the so called attributable risk. Clearly, $\tilde{r_{\text{xy}}}<\hat{r_{\text{xy}}}<r_{\text{xy}}$.

Just as for $\hat{r_{\text{xy}}}$, no method can accurately identify all reports corresponding to $\tilde{Y}$ events. Experienced pharmacovigilance experts should be able to assess all $N_{\text{xy}}^{R}$ reports at hand and based on the reported information decide which reports to include for the upper and lower limit, repsectively. However, to thoroughly outline such a process is well beyond the scope of this paper [23]. A conservative automated approach might be to select for the upper limit all reports that have some reported information suggestive of *X* as a cause, e.g. a positive rechallenge reaction, a plausible time to onset, absence of co-suspected drugs, a high degree of reported suspicion for a causal link, or – for some adverse effects – a positive dechallenge reaction; whereas for the lower limit several of these features are required simultaneously in order for a report to be selected.

### Using a restricted time to event onset

Many adverse effects of interest occur within a relatively limited time from start of drug exposure. This is typically true for those rare and idiopathic adverse effects that are difficult to study outside of spontaneous reporting systems, so called type B effects [24]. If it is possible to assign a time *t* from start of exposure to *X* to onset of *Y* that will capture more or less all *Y* events attributable to *X*, this is generally adviseable to do. This is true even if one considers total rather than attributable risk, since there is little value in covering time during which all events are likely due to the general background. Clearly this general advise can be overruled if the investigated risk is part of a larger analysis with a set follow-up time.

The main practical issue is that the time to onset may not be calculable from the information provided on the reports. If this information is missing for a substantial proportion of the $N_{\text{xy}}^{R}$ reports at hand this may threaten the assumption regarding relative over-reporting, and therefore the validity of the upper limit. A pragmatic solution is to conservatively include all reports with missing information on time to onset into $N_{\text{xy}}^{R}$ for the calculation of *ρ*_*xy*_, thereby not jeopardising its validity as an upper limit.

### Probabilistic analysis

In some settings it may not suffice with merely an interval that contains *r*_*xy*_. One example is probabilistic benefit-risk assessment [25,26].

There is nothing inherent to this framework that predicts where within the interval that *r*_*xy *_is more likely to fall, and also there is very limited past experience on which to base an empirical guess. Therefore probabilistic analyses present a significant challenge, and we can only reason generally around possible solutions.

The coarsest approach is to completely avoid any probabilistic use of the intervals, and proceed to a best case/worst case analysis. In a benefit-risk assessment one would then impute first all lower and then all upper limits computed for *X*, to be combined with the other, probabilistic, data. This would then yield two different results, which, if different, would preclude any conclusion from the assessment. Indeed, this is the only possible method in benefit-risk assessment based on point values [27].

**Figure 4:**
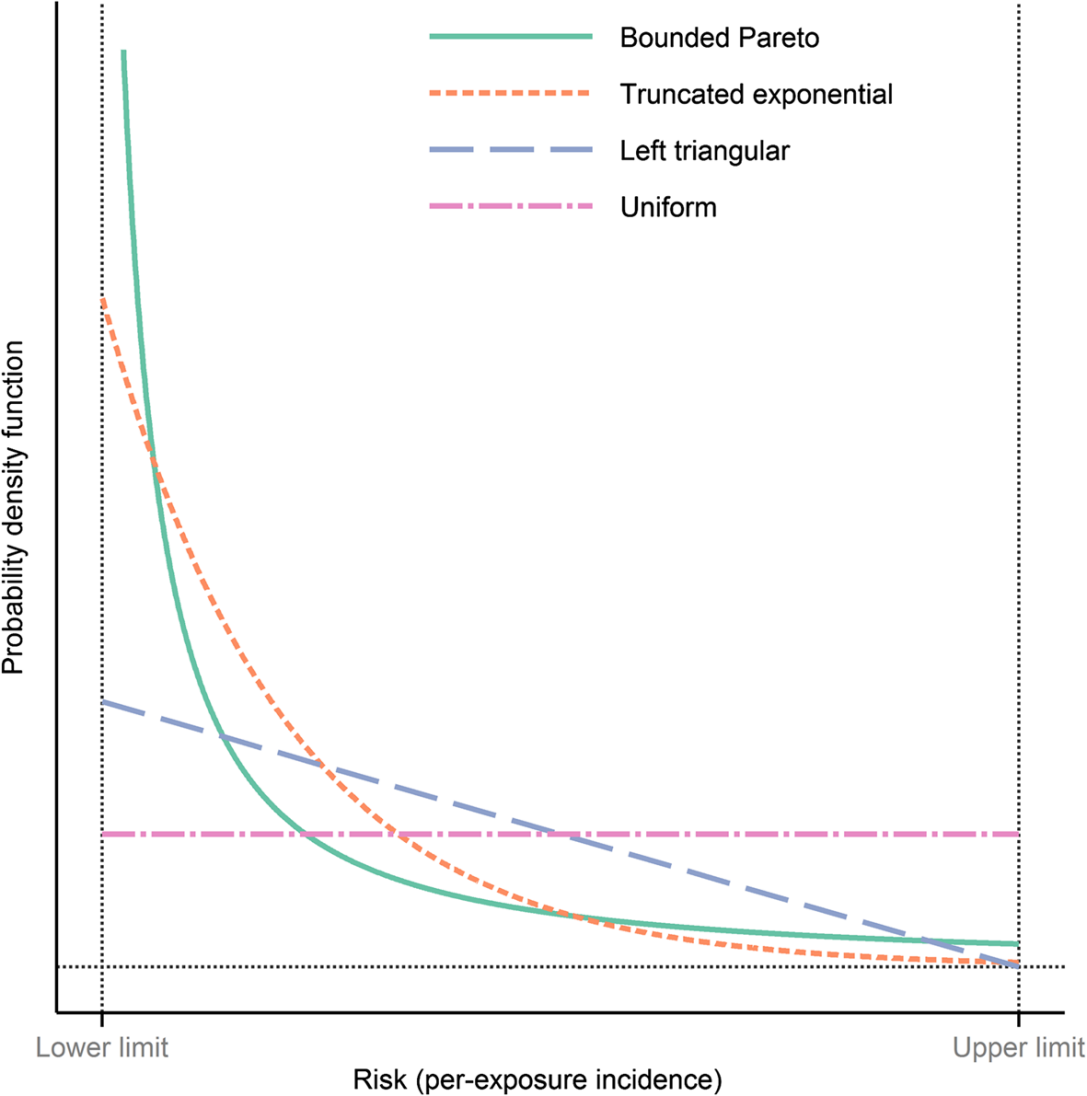
**Examples of probability distributions to use over risk intervals in probabilistic analyses. **In this example the lower limit is 0.03% and the upper limit 1%. The bounded Pareto distribution has a scale parameter of 0.25 and the exponential distribution has a rate parameter of 5/(Upper limit-Lower limit) before truncation. The uniform distribution corresponds to equal belief in all risks between the lower and upper limits. In contrast, the triangular distribution with mode at the lower limit puts more density on lower risks, but is still fairly likely to yield high values. Both the bounded Pareto and the truncated exponential clearly favour lower risks. Their main difference is that the former corresponds to stronger belief in risks close to both the lower and the upper limit. Note that to benefit the clarity of the display, the graph for the bounded Pareto distribution has been truncated. In reality it extends much higher for risks close to the lower limit.

A more sophisticated approach is to carry out a sensitivity analysis over different types of probability distributions. There may be some external information that makes either end of the interval more likely; for example, based on the number of patients studied in clinical trials, the adverse effect should have been highly probable to appear in those trials if the true risk were on the upper half of the interval. If so, one can start from the uniform distribution and proceed towards distributions more and more skewed towards the lower limit. Figure 4 provides a few reasonable examples.

### Estimating drug exposure

Within this framework, the calculation of a lower limit for *r*_*xy *_involves an estimate of $N_{x}^{E}$, i.e. the number of exposures to *X*. This implies that some data source external to the database of individual case reports is required.

One alternative is to use sales data for this purpose, which is an old idea in pharmacovigilance [28]. However, depending on the variability in dose and duration for the drug of interest, quite strong assumptions may be needed to convert aggregate sales quantities into numbers of exposures as defined here.

Other alternatives are to use prescription registries or databases of electronic health records [29,30]. Both these types of data sources follow individual patients over time, which enables accurate estimation of exposure. Their main drawback in this context is that they solely cover prescription drugs.

### Uncertainty related to small counts

The framework presented here differs from most epidemiological methods in that it is not based on point estimation of some effect parameter of interest combined with hypothesis tests or uncertainty intervals rooted in statistical theory. Rather, because we make observations not in the actual population of treated patients but in a connected population of case reports, point estimation of risk is not feasible.

It may be intuitively difficult to accept intervals based on very low values of $N_{\text{xy}}^{R}$, as in the example with the Finnish data for the Pandemrix-narcolepsy association. As mentioned in Section ‘General validity of the model’, with small counts one needs to be extra careful in considering whether each report corresponds to an actual adverse episode. The assumption of relative over-reporting, however, is not per se threatened by small counts. In particular, if *Y* is very rare it can be expected to be reported with *X* only a small number of times, even if there is a connection between *X* and *Y*.

Our intervals provide deterministic limits for the incidence $N_{\text{xy}}^{E}/N_{x}^{E}$ in the sense that they follow logically from a set of assumptions. It should be noted that there is no sampling involved with respect to $N_{\text{xy}}^{E}$ and $N_{x}^{E}$ even though these are not necessarily known, and consequently there is no sampling variability for the limits on the risk that applies to the $N_{x}^{E}$ exposures that have already occurred. While such uncertainty does apply to future exposures, we view this as a generally negligible threat to the validity of our limits: $N_{x}^{E}$ is typically large, and, as seen in Equation 4, the upper limit has an extra margin of error by a factor $N_{\text{xy}}^{E}/N_{\text{xy}}^{A}$. The possible exception when this effect may need to be considered for the risk in future exposures is when *X* is a very rarely used drug.

## Limitations and future directions

A key next step is to complement the modest empirical validation of the suggested approach provided in this article. Two isolated examples can serve mainly as an indication that the theoretical reasoning is correct. On the other hand, the derived upper and lower limits follow logically from a set of assumptions that have been thoroughly discussed. This should allow any potential user to judge whether or not this approach is suitable for the particular problem (s)he is facing.

The empirical validations that consequently should follow are indeed challenging. A major difficulty lies in determining what the true risk is, which is a prerequisite for judging whether the computed risk limits are satisfactory. This is challenging for several reasons: one needs to identify a data source that covers the same population as one’s database of individual case reports; one needs to reasonably transfer the exposure definition to that setting; and one needs to define the adverse effect of interest within the medical terminology used in that particular data source. The issue is further complicated by the fact that no data source is likely to have perfect capture of medical events, and therefore in a sense the ‘true’ risks are likely to be lower limits as well.

For the combination of prescription drugs and adverse events that require medical attention, the most useful data source seems to be databases of electronic health records. Larger controlled trials are another alternative, especially if conducted in heterogeneous patient populations that reasonably resemble clinical reality. Naturally only such adverse effects can be used for validation that are quantifiable in another data source; these are, however, not of primary interest in practice. It may also be possible to consult the literature for a collection of true risks against which to compare. However, most epidemiological studies report measures of relative rather than absolute risks.

We believe that a proper validation should not only compare the computed risk limits with some reference value of true risk, but it should also investigate the underlying assumptions directly. Adding those two aspects together should yield very useful information on the circumstances under which this framework could be expected to work, and on any general tendencies in the relationship between the computed risk limits and the true risk.

## Conclusions

This paper presents a conceptual model that links collections of individual case reports to drug exposures and occurrences of adverse events in the real world. Based on this model, necessary assumptions are derived that permit reporting ratios to be used as upper limits for risks in the real world. It is also shown how and when report counts can be combined with external estimates of drug exposure to construct lower risk limits, and the entire framework is applied to data from two real examples with satisfactory results.

Our in-depth discussion of the underlying assumptions shows that the framework is best suited for serious adverse effects, which will typically be those rare effects for which quantification by other means is most difficult. While this discussion also shows that short duration of treatment and a healthy patient population are other factors that favour the validity of the approach, practical countermeasures are presented for other scenarios.

This work will offer a much needed alternative in any quantitative analysis that involves drug risks. It will be particularly useful in structured benefit-risk assessments that include rare adverse effects that cannot be otherwise quantified without retreating to entirely subjective guesswork. Not only are such assessments right on the path along which pharmacovigilance and medicines regulation in general are moving, but already today they are part of regulatory post-marketing guidelines adopted globally.

## Endnote

^a ^Both Finland and Sweden belong to the 117 members of the WHO Programme for International Drug Monitoring, and therefore forward their individual case reports to VigiBase. However, to avoid the risk of misrepresenting their respective national data on this sensitive issue, we chose to contact these centres directly and use their locally recorded information.

## Competing interests

The authors declare that they have no competing interests.

## Authors’ contributions

OC developed the model and derived the equations that follow from it, collected and analysed the data on the two examples, and drafted the manuscript. GNN and IRE critically reviewed the draft manuscript. All authors read and approved the final manuscript.
